# Detection of Building Equipment from Mobile Laser Scanning Point Clouds Using Reflection Intensity Correction for Detailed BIM Generation †

**DOI:** 10.3390/s25226937

**Published:** 2025-11-13

**Authors:** Tomohiro Mizoguchi

**Affiliations:** Department of Informatics and Data Science, Faculty of Engineering, Sanyo-Onoda City University, Sanyo-Onoda 756-0884, Japan; mizoguchi@rs.socu.ac.jp

**Keywords:** laser scanning, point cloud, 3D modeling, building equipment, building information model

## Abstract

**Highlights:**

**What are the main findings?**

- Accurate automatic extraction of building equipment using corrected reflectance intensity from MLS point clouds
- Capable of detecting even small and flat objects with almost no omission

**What is the implication of the main finding?**

- Use of corrected laser intensity is effective for detailed BIM reconstruction from point clouds including building equipment

**Abstract:**

The Building Information Model (BIM) has been increasingly adopted for building maintenance and management. For existing buildings lacking prior digital models, a BIM is often generated from 3D scanned point clouds. In recent years, the automatic construction of simple BIMs comprising major structural elements, such as floors, walls, ceilings, and columns, has become feasible. However, the automated generation of detailed BIMs that incorporate building equipment, such as electrical installations and safety systems, remains a significant challenge, despite their essential role in facility maintenance. This process not only enriches the information content of the BIM but also provides a foundation for evaluating building safety and hazard levels, as well as for supporting evacuation planning and disaster-preparedness simulations. Such equipment is typically attached to ceilings or walls and is difficult to detect due to its small surface area and thin geometric profile. This paper proposes a method for detecting building equipment based on laser reflection intensity, with the objective of facilitating the automatic construction of detailed BIMs from point clouds acquired by mobile laser scanners (MLSs). The proposed approach first corrects the reflection intensity by eliminating the effects of distance and incidence angle using polynomial approximation, thereby normalizing the intensity values for surfaces composed of identical materials. Given that the corrected intensity approximately follows a normal distribution, outliers are extracted as candidate points for building equipment via thresholding. Subsequently, the point cloud is converted into a 2D image representation, and equipment regions are extracted using morphological operations and connected component labeling. Experiments conducted on point clouds of building ceilings and walls demonstrate that the proposed method achieves a high detection accuracy for various types of building equipment.

## 1. Introduction

**Figure 1:**
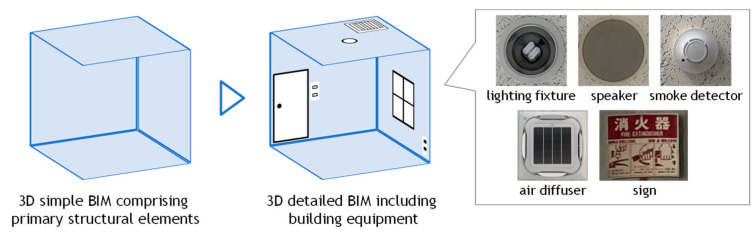
Construction of detailed BIM from point cloud.

The proper use of the Building Information Model (BIM) throughout a building’s lifecycle can result in substantial cost savings for facility managers and owners. However, as 3D models are typically unavailable for existing or older buildings, the BIM is often generated from 3D scanned point clouds. Over the past decade, significant research and development efforts have focused on the automatic generation of BIM from such point clouds. As a result, it is now feasible to automatically construct basic BIMs comprising primary structural elements such as floors, walls, ceilings, and columns [1,2,3]. However, as illustrated in Figure 1, the automatic generation of detailed BIMs which include, for example, electrical equipment such as lights, speakers, and switches, and safety equipment such as evacuation signs, fire alarms, and sprinklers essential for maintenance and facility management remains an unsolved problem. In the field of safety and disaster prevention, detailed BIMs that integrate electrical and safety equipment can support risk assessment of fire, power outage, or structural hazards and can be directly utilized for evacuation planning and disaster-response simulations. In terms of facility operation and maintenance, such enriched BIMs facilitate the scheduling of equipment inspections, the planning of replacements or upgrades, and the management of lifecycle costs, thereby improving both operational efficiency and long-term reliability. However, these components are typically attached to ceilings or walls and are characterized by small surface areas and minimal thickness. As such, they are difficult to detect based solely on geometric features of the point cloud [4]. To address this limitation, it is necessary to develop methods that utilize non-geometric information for the detection of such building equipment, thereby enabling the integration of these elements into basic BIMs and facilitating the generation of detailed BIMs.

In this paper, we propose a method for detecting building equipment using laser reflection intensity contained in point clouds acquired by mobile laser scanners (MLSs), with the aim of automatically reconstructing detailed BIM. Compared to conventional terrestrial laser scanners (TLSs), an MLS offers the advantage of efficiently scanning wide areas and has seen increasing adoption in recent years [5]. Laser reflection intensity represents the strength of the laser signal reflected from a target surface. As it varies depending on the target’s color and material, it is considered highly effective for detecting building equipment within point cloud data. However, the raw reflection intensity obtained from the scanner is affected by both the scanning distance and the angle of incidence. Therefore, appropriate correction is required to isolate the component of intensity that reflects only material and color differences. Typically, ceilings and walls are flat, expansive surfaces composed of uniform materials, onto which building equipment—often made of different materials and colors—is installed. Based on this observation, the fundamental idea of the proposed method is as follows: by correcting the reflection intensity of ceilings and walls such that it approximates a constant value, regions exhibiting significantly different intensity values can be effectively identified as potential building equipment.

Object recognition in point clouds typically consists of two main steps. The first is the extraction step, in which partial point clouds corresponding to individual objects are segmented. The second is the classification step, where each extracted object is assigned a label (e.g., lighting fixture, fire alarm). This study focuses solely on the first step—object extraction. We posit that, once reliable extraction is achieved, high-accuracy classification can subsequently be performed by applying AI-based methods to corresponding partial image representations of each object, using appropriate image processing techniques.

## 2. Related Works

### 2.1. Building Equipment Detection from Point Cloud for Detailed BIM Reconstruction

BIM reconstruction from scanned point clouds has been extensively studied [2,3]. In previous work, the fundamental idea was to extract planar elements such as floors, walls, ceilings, columns, and stairs using geometry processing techniques, and then construct a 3D model from their combinations. Representative approaches include region growing [6] and RANSAC-based algorithms [7,8]. In the field of computer graphics, methods have also been proposed to identify the optimal combination from a large set of candidate planes extracted from low-quality point clouds and to regularly align them by solving complex optimization problems [9,10]. As a result of these advances, it has become possible to automatically generate basic BIMs comprising the primary structural components described above.

As a next step, research has focused on enriching the informational content of basic BIMs by adding attribute information, thereby increasing their level of detail. For example, in the study by Hensel et al. [11], images of building facades were analyzed to extract information of regularly arranged rectangular shapes, such as windows and doors. Furthermore, by aligning these attributes according to predefined rules and incorporating them into a LoD2 model, the level of detail of the reconstructed model was enhanced to LoD3. Pantoja-Rosero et al. [12] constructed a 3D point cloud from multiple images using Structure from Motion (SfM) and generated a LoD2 model based on planar sets. Furthermore, they extracted openings such as windows and doors from the images and incorporated them into the LoD2 model, thereby enhancing the level of detail to LoD3. Moreover, Pantoja-Rosero et al. [13] also extracted wall cracks and integrated them into the model, further enriching the reconstructed model.

With the increasing research and development efforts aimed at enhancing the level of detail in BIM, recent studies have increasingly focused on the automatic detection of small-scale building equipment and the construction of detailed BIMs that incorporate these elements. Akiyama et al. [14] proposed a method for detecting building equipment on ceiling surfaces using point clouds obtained from terrestrial laser scanning (TLS). In the preprocessing stage, 2D ceiling regions are extracted and structured from the point cloud data. Subsequently, partial point clouds corresponding to individual equipment, such as lighting and air conditioning units, are identified, and geometric primitives (circles and rectangles) are fitted to their boundary points. However, detecting objects with small thickness is challenging when relying solely on geometric information. Furthermore, the detection rate significantly decreases when the scanning distance exceeds 5 m, necessitating an increased number of scans for larger areas. It should be noted that classification processing was not addressed in this study.

In recent years, the development of convolutional neural networks (CNNs) has advanced, and they have been increasingly applied to a wide range of tasks in image processing [15] and point cloud processing [16,17], including classification, object detection, and semantic segmentation. Several studies have also reported the application of CNNs to the extraction of small-scale equipment from images and point clouds for the purpose of constructing detailed BIMs. For example, Pan et al. [18] proposed a method for detecting building equipment using both images and point clouds. Specifically, two 3D point clouds, one generated via Structure-from-Motion and Multi-View Stereo (SfM-MVS) and the other via laser scanning, are first acquired and then merged. In parallel, building equipment is detected and classified using a convolutional neural network (CNN) applied to video data, and the identified objects are integrated into the point cloud to construct detailed BIM. However, since this approach relies on the fusion of two types of point cloud data, it involves complex processing and is time-consuming. Moreover, while CNN-based object detection is effective for identifying equipment with limited shape variation (e.g., fire extinguishers and fire alarms), it requires large amounts of training data for objects with greater shape diversity. Geyter et al. [19] employed Indoor Mobile Mapping (IMM) to acquire point clouds and panoramic images of building interiors. In their approach, panoramic images were divided into small regions, and object detection was performed individually for each region. Detected objects were then projected onto the point cloud using camera position and orientation data. However, this method also faces challenges in obtaining sufficient training data for CNN-based detection. Anjanappa et al. [20] also utilized CNN on 3D point clouds to detect and classify building equipment within indoor environments. However, as with the methods, a substantial amount of annotated training data is required to accurately detect the wide variety of building equipment types.

### 2.2. Laser Intensity Correction

Point cloud data acquired by a laser scanner contain not only three-dimensional coordinates (x,y,z), but also the intensity information corresponding to each scanned point. Intensity represents the strength of the signal returned to the receiver when a laser pulse is emitted onto the surface of an object, and it indirectly reflects the reflective properties and surface conditions of the object. Consequently, the material, color, and surface roughness of the object have a significant influence on the intensity values. For example, metals and painted surfaces generally exhibit strong reflections, whereas asphalt and vegetation tend to produce weaker reflections. Taking advantage of this property, many studies have utilized intensity information for object recognition in various environments. Specifically, applications such as road marking detection [21,22], vehicle detection [23], building window detection [24], construction material classification [25], and even the segmentation of tree trunks and leaves [26] have been reported.

The reflectance intensity values obtained from a laser scanner are generally influenced not only by the reflectivity of the target surface, but also by factors such as distance, incident angle, sensor characteristics, and atmospheric conditions. Therefore, it is not recommended to use these values directly without appropriate correction [27].

However, when scanning is performed using a single scanner over a short period under consistent environmental conditions, the effects of sensor characteristics and atmospheric conditions can be considered negligible. In such cases, the variation in reflectance intensity can be attributed primarily to the target’s reflectivity, the incident angle, and the distance, as described by the radar equation. Many experimental studies have been conducted to investigate the range and incidence-angle effects and to develop correction methods for single-wavelength LiDAR systems. Kaasalainen et al. [28] presented one of the earliest systematic frameworks for separating and correcting the range and incidence-angle effects in terrestrial laser scanner (TLS) intensity calibration, which has been regarded as an early and influential study that inspired subsequent experimental and theoretical research [27,29,30]. It is now theoretically demonstrated based on the LiDAR equation that the three components of range, incidence angle, and reflectance can be independently separated and modeled, as expressed in Equation (1) [27,28,29,30,31].

(1) $$I\left(\rho ,\theta ,d\right)=f_{1}\left(\rho \right)\cdot f_{2}\left(\theta \right)\cdot f_{3}\left(d\right).$$

Here, *I*(*ρ*,*θ*,*d*) denotes the initial reflection intensity, and *f*_1_, *f*_2_, and *f*_3_ represent the functions of the target reflectance *ρ*, incident angle *θ*, and distance *d*, respectively.

The independence of the range and incidence-angle terms in Equation (1) has been theoretically discussed based on an anisotropic bidirectional reflectance LiDAR model. Bai et al. [32] showed that the distance effect is wavelength-independent and instrument-dependent, whereas the incidence-angle effect is wavelength-dependent and governed by the target’s backscattering characteristics. This analysis provided a theoretical basis for treating range and angular effects separately in LiDAR intensity correction.

Theoretically, the reflection intensity is directly proportional to the cosine of the incident angle and inversely proportional to the square of the distance. However, due to the complex nature of the effects caused by distance and incident angle, it has been shown that correcting these effects using a polynomial approximation, as expressed in Equation (2), is effective.

(2) $$I_{cor}\left(\rho \right)=I\left(\rho ,\theta ,d\right)\cdot \frac{\sum_{i=0}^{N_{2}}\left(\alpha_{i}{\theta_{s}}^{i}\right)}{\sum_{i=0}^{N_{2}}\left(\alpha_{i}\theta^{i}\right)}\cdot \frac{\sum_{i=0}^{N_{3}}\left(\beta_{i}{d_{s}}^{i}\right)}{\sum_{i=0}^{N_{3}}\left(\beta_{i}d^{i}\right)},$$

where *α_i_* and *β_i_* are polynomial coefficients, and *N*_2_ and *N*_3_ represent the degrees of the polynomial with respect to distance and incident angle, respectively. To estimate these coefficients, it is necessary to derive the relationships between the reflection intensity and both distance and incident angle, and to define appropriate correction equations. There have been several studies on reflection intensity correction targeting 2D LiDAR [31] and 3D laser scanners.

One common approach to deriving a correction equation involves using a physical target to empirically determine the relationships between reflection intensity, distance, and incident angle. In the method proposed by Tan and Cheng [29], a terrestrial laser scanner (TLS) was used to scan a target multiple times in a controlled laboratory environment from various distances and angles. Polynomial correction functions were then derived based on the measured reflection intensity values. Specifically, the target was first scanned from a fixed distance while varying the incident angle from 0° to 90° at small intervals (e.g., 5° increments), yielding a set of angle–intensity samples. Similarly, scans were performed at fixed angular positions while varying the distance in constant intervals, resulting in a set of distance–intensity samples. By applying least-squares fitting to these datasets, polynomial correction equations were obtained to model the relationships between incident angle and reflection intensity, as well as between distance and reflection intensity. However, modeling the distance–intensity relationship is more complex than that of the incident angle, as it requires fine-resolution scanning over distances of several tens of meters. This results in significant experimental overhead in terms of time and effort. To address this issue, Tan and Cheng [30] later proposed a method for estimating the correction equation for distance and reflection intensity directly from real-world scanning point cloud data. In this approach, the angle correction is first applied using the correction equation derived from the target-based calibration, as described above and shown in Equation (3).

(3) $$I_{\theta }\left(\rho ,d\right)=I\left(\rho ,\theta ,d\right)\cdot \frac{\sum_{i=0}^{N_{2}}\left(\alpha_{i}{\theta_{s}}^{i}\right)}{\sum_{i=0}^{N_{2}}\left(\alpha_{i}\theta^{i}\right)}.$$

The incident angle–corrected reflection intensity, denoted as *I_θ_*(*ρ*,*d*), represents a reflection intensity influenced solely by the distance. Therefore, the correction equation for the distance–reflection intensity relationship can be derived by applying least-squares regression to the intensity values after incident angle correction. The final corrected reflection intensity, *I_cor_*(*ρ*), can then be computed using Equation (2), based on the estimated polynomial parameters.

In addition to the polynomial-based correction method described above, a look-up table (LUT)—based approach has also been proposed by Jeong and Kim [33]. This method is based on the observation that, for two scanned surfaces at the same distance and incident angle, the ratio of their reflection intensities should correspond to the ratio of their reflectance values. The LUT is constructed as a two-dimensional grid, with distance on the vertical axis and incident angle on the horizontal axis. For each cell in the grid, the mean and standard deviation of the reflection intensity are calculated using sample data corresponding to that specific distance and angle. To obtain this data, a reference surface is used—typically a large, planar area composed of uniform material, such as an exterior wall of buildings several meters in size. Using this LUT, the reflectance corresponding to a given reflection intensity can be estimated, as described in Equation (4).

(4) $$I_{cor}\left(\rho \right)\propto I\left(\rho_{ref}\right)\cdot \frac{I\left(\rho ,\theta ,d\right)}{I\left(\rho_{s},\theta ,d\right)},$$

where the reflection intensity *I*(*ρ_s_*,*θ*,*d*) of the reference surface is defined as the average reflection intensity corresponding to each cell in the LUT. Since the true reflectance *I*(*ρ_ref_*) of the reference surface is unknown, it is arbitrarily assigned a constant value.

In contrast to the aforementioned methods, an alternative approach has been proposed that derives the correction equation directly from the acquired scanning point cloud, without the need for a pre-defined correction equation or look-up table (LUT). For example, in road surface scanning using vehicle-mounted LiDAR, if the height between the scanner and the road surface remains constant, the incident angle can be expressed as a function of distance. Therefore, as shown in Equation (5), correcting for distance alone can simultaneously account for the effect of the incident angle [34].

(5) $$I_{cor}\left(\rho \right)=I\left(\rho ,\theta ,d\right)\cdot \frac{\sum_{i=0}^{N_{3}}\left(\beta_{i}d_{s}^{i}\right)}{\sum_{i=0}^{N_{3}}\left(\beta_{i}d^{i}\right)}.$$

The effectiveness of the proposed reflection intensity correction method was verified through experiments involving the extraction of road markings from MLS point clouds. Although this approach requires specific range conditions, it enables correction using only the point cloud data, making it applicable to a wide range of scenarios.

These practical approaches to reflection intensity correction are fundamentally based on empirical observations of the relationships among distance, incident angle, and reflectance.

## 3. Method

### 3.1. Overview of the Proposed Method

**Figure 2:**
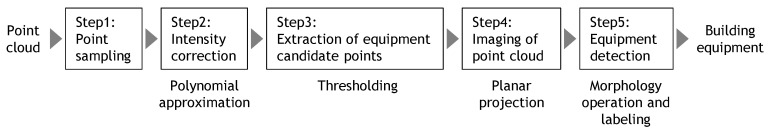
Overview of the proposed method.

An overview of the proposed method is shown in Figure 2. In this method, the target region is first manually extracted, and preprocessing is applied to sample the point cloud so that the point density becomes spatially uniform. Subsequently, the effects of distance and incident angle on the reflection intensity are corrected using polynomial approximation, and the reflection intensity values of walls and ceilings composed of the same material are adjusted to approximate a constant value. Next, assuming that the corrected reflection intensity follows a normal distribution, points that significantly deviate from this distribution are identified as equipment candidates through a thresholding process. The point cloud is then converted into a binary image, and individual equipment components are extracted using morphological operations and connected-component labeling. In this study, we focus on regions scanned under conditions where the distance between the scanner and the target surface is kept constant. Further methodological details are provided in the following sections.

### 3.2. Point Sampling

The point cloud acquired from the scanner is high-density data that includes not only the target object but also its surrounding environment. As the scanning area increases, the volume of data can become enormous, often reaching tens to hundreds of millions of points. To facilitate efficient handling and processing, we utilize the point cloud processing software Cloud Compare v2 to extract the target region and down sample the point cloud at 1 cm intervals.

### 3.3. Intensity Correction

In this method, distance and incident angle corrections are simultaneously performed using the approach proposed by Wan et al. [34], as introduced in Section 2.2. This method has the advantage that it does not require prior experiments to construct a correction model, and the correction process can be executed immediately once point cloud data are obtained. Furthermore, compared with other correction methods, it is simpler to implement and is considered effective for BIM construction.

In the correction process, a scatter plot was generated from the scanning point cloud, with range on the horizontal axis and reflection intensity on the vertical axis. A polynomial curve was then fitted to the data points using the least-squares method. In this study, all points from the scan were included in the fitting process. Ideally, points corresponding to building equipment should be excluded from the fitting, and a robust fitting method incorporating outlier removal or weighted regression would be preferable, such as RANSAC or Huber loss, to reduce the influences of outliers. However, as discussed later, the results indicate that a conventional least-squares approach yields sufficient correction accuracy for the purpose of this study. The fitting procedure was implemented based on the polynomial fitting and least-squares regression source codes provided in Numerical Recipes [35].

**Figure 3:**
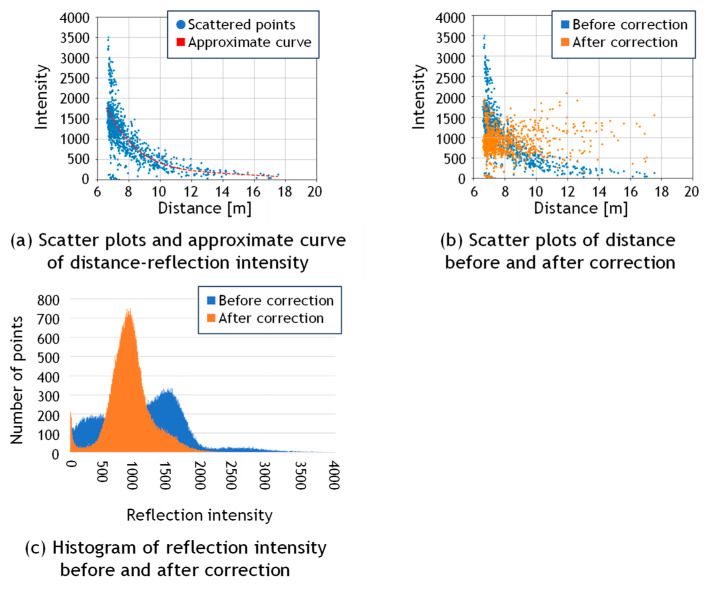
Reflection intensity correction.

Figure 3a shows the scatter plot of distance versus reflection intensity, along with the fitted correction curve. In this graph, only 500 points are randomly displayed for better visibility. From this graph, it can be observed that the intensity values decrease monotonically as the scanning distance increases. Therefore, following the practice of Wan et al. [34], the degree of the polynomial was set to *N*_3_ = 3, and the value of *d*_s_ in Equation (5) was set to the average scanning distance for all the examples in this paper. A simple preliminary experiment was conducted regarding the polynomial degree, and it was confirmed that increasing the degree beyond three resulted in almost no reduction in residuals. The pseudocode describing this procedure is presented in Algorithm 1.
**Algorithm 1.** Intensity Correction by Polynomial FittingInput: A set of scattered 2D points $\left\{d_{i},I_{i}\right\}$, where $d_{i}$ is the scanning distance and $I_{i}$ is the raw intensity of each scanned point $p_{i}$. Fit polynomial function $f\left(d\right)=a_{0}+a_{1}d+a_{2}d^{2}+\cdots +a_{N}d^{N}$ to the scattered 2D points using least-squares method minimizing $\sum_{i=1}^{n}{\left(f\left(d_{i}\right)-I_{i}\right)}^{2}$, where *N* is the degree of polynomial and *n* is the number of total points. We set *N* = 3 for all the examples in this paper.Compute the averaged distance $d_{s}$ as $\frac{1}{n}\sum_{i=1}^{n}d_{i}$.Compute corrected intensity of each point $p_{i}$ as $I_{i}^{cor}=I_{i}\frac{f\left(d_{s}\right)}{f\left(d_{i}\right)}$ following Equation (5).
Output: Corrected intensity of each point $I_{i}^{cor}$.

**Figure 4:**
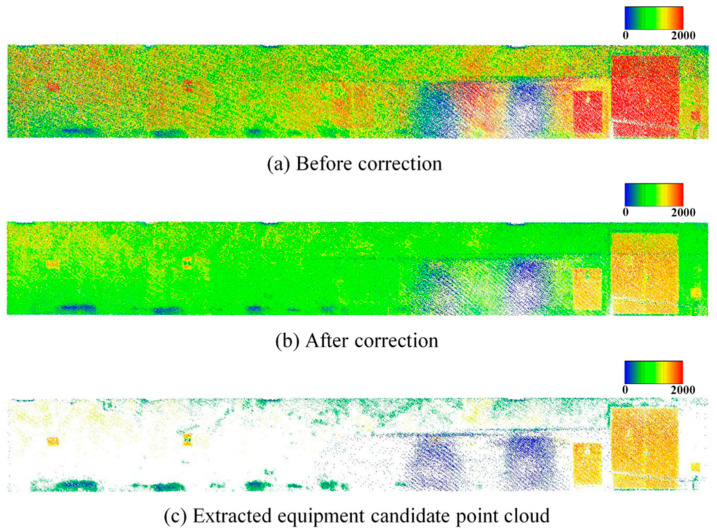
Results of reflection intensity correction on point cloud.

Figure 3b presents the sample points and Figure 3c shows the histogram of reflection intensity values before and after correction. It can be observed that the reflection intensity values, which originally decreased with increasing distance, are corrected to approximate a constant value regardless of distance. Furthermore, it indicates that the corrected values approximately follow a normal distribution. This trend is also visually confirmed in the point clouds shown in Figure 4a,b, which display the data before and after correction, respectively. The data was acquired by scanning the walls of an underground parking lot. Before correction, considerable color variations corresponding to fluctuations in reflection intensity were observed in the background wall regions; however, after correction, the color became nearly uniform, confirming that the correction brought the intensity values closer to a constant level. This is likely because the reflection intensities of points corresponding to large, uniform surfaces composed of the same material were properly normalized.

### 3.4. Extraction of Equipment Candidate Points

Based on the normalized and corrected reflection intensity, it is assumed that points corresponding to walls and ceilings lie within a certain range around the mean intensity value, while points corresponding to building equipment deviate from this range. Therefore, candidate points for building equipment are extracted using a thresholding process based on the average *μ* and standard deviation *σ* of the reflection intensity. The threshold is defined as *th* = *μ* ± *ασ*, where *α* is a user-defined parameter. Figure 4c presents an example of the point cloud containing the extracted equipment candidates. It can be confirmed that the majority of the wall regions were appropriately excluded.

**Figure 5:**
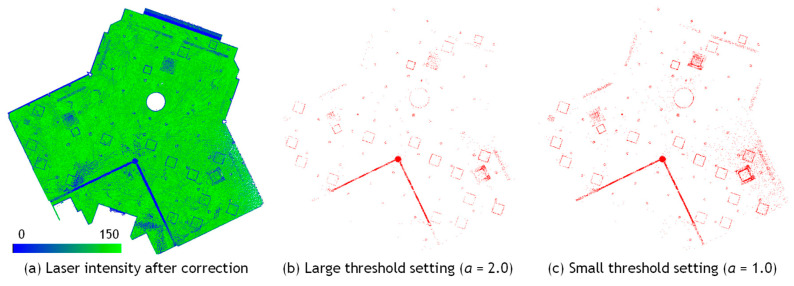
Comparison of threshold setting for equipment candidate detection.

Figure 5 shows a comparison of the detection results of equipment candidate points under different threshold settings. Figure 5a presents an example of the reflection intensity correction result obtained by applying the procedure described in the previous section to the MLS point cloud of a building ceiling surface. As shown in Figure 5b, when the threshold parameter α is set to a large value, the number of points extracted as potential equipment candidates decreases, while false detections of non-equipment regions are also reduced. Conversely, as shown in Figure 5c, when α is set to a smaller value, equipment objects become easier to detect, but the number of false detections of non-equipment regions increases. This threshold is defined as a multiple of the standard deviation of the corrected intensity values and is independent of the manufacturer’s internal scaling of intensity data. Therefore, it can be applied consistently across different scanner models. However, since the correction performance varies slightly among scanning and the intensity range of actual equipment cannot be determined without examining the data, the appropriate α value was determined empirically by visually inspecting the detection results. In general, satisfactory performance was obtained with *α* = 1.0 or *α* = 2.0, indicating that precise parameter tuning was not required.

### 3.5. Imaging of Point Cloud

From the equipment candidate point cloud obtained in the previous step, the points are grouped according to individual equipment instances. First, the candidate point cloud is projected onto the least squares planes corresponding to walls or ceilings. The least-squares plane was determined by fitting to the set of points distributed on planar surfaces corresponding to walls or ceilings. The projection direction was defined along the normal vector of the fitted plane. The proposed method does not perform strict geometric evaluation of detailed shapes such as circles or rectangles. Instead, after clustering and morphological processing, detection is conducted based on the presence or absence of pixels in the projected image. Although minor geometric distortions may occur due to the planar projection, these distortions do not lead to a decrease in detection accuracy.

The projection plane is then divided into a grid to generate a binary image. The grid size must be set sufficiently small to ensure that small target objects are represented by an adequate number of pixels. In the dataset used in this study, objects with a diameter of approximately 10 cm are present. Therefore, the grid size was set to 2 cm, which allows such small objects to be represented by about 20 pixels, providing sufficient resolution for reliable extraction.

**Figure 6:**
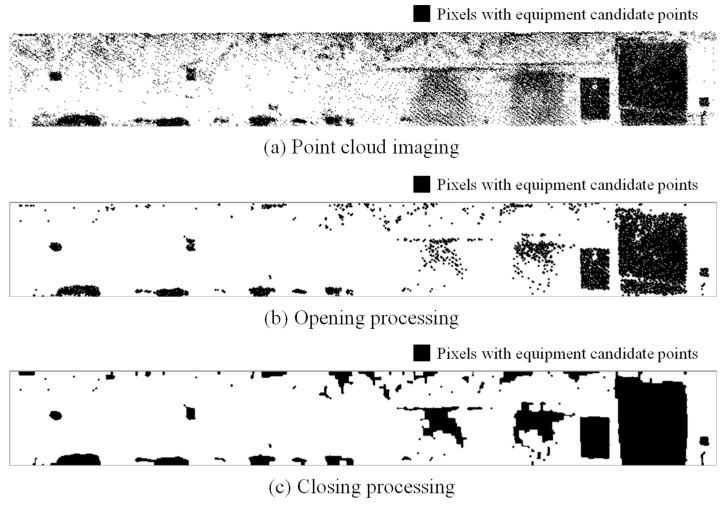
Morphological process.

An example of the resulting image is shown in Figure 6a. In the binary image, pixels containing one or more projected points are rendered in black, while all other pixels are rendered in white.

### 3.6. Equipment Detection

From the image obtained through the process described in the previous section, it can be observed that some pixels unrelated to actual equipment have been incorrectly extracted, and that certain regions corresponding to a single piece of equipment have been fragmented. To address these issues, morphological processing is applied to the binary image. Morphological operations consist of dilation, which expands binary regions by one pixel, and erosion, which contracts them. In the opening operation, erosion is performed *n* times, followed by *n* iterations of dilation. This process is effective for removing isolated pixels. However, if the number of iterations is too large, it may result in the fragmentation of a single equipment object into multiple parts or the removal of small equipment elements. Conversely, the closing operation performs *n* iterations of dilation followed by *n* iterations of erosion. This operation helps close small holes and connect disjointed components in the binary image. However, excessive iterations may cause adjacent equipment objects to merge into a single region. Therefore, the number of iterations *n* was empirically determined based on the shape and size of the equipment to be extracted. In this study, the number of iterations *n* was set between 1 and 4, depending on the dataset. Figure 6b,c show the results after applying the opening and closing operations, respectively. It can be observed that the opening operation removes isolated pixels, while the closing operation reconnects fragmented components. Finally, connected-component labeling is applied to assign unique labels to each connected region in the binary image, enabling the extraction of individual equipment objects. These labels are also assigned to the corresponding point cloud to preserve the link between image and 3D data.

## 4. Results and Discussion

### 4.1. Test Site and Scanners

The proposed method was applied to point cloud datasets acquired using two different scanners: the Ouster OS0 and the Velodyne VLP-16. The specifications of each scanner are summarized in Table 1. Table 2 shows the summary of our test site.

**Figure 7:**
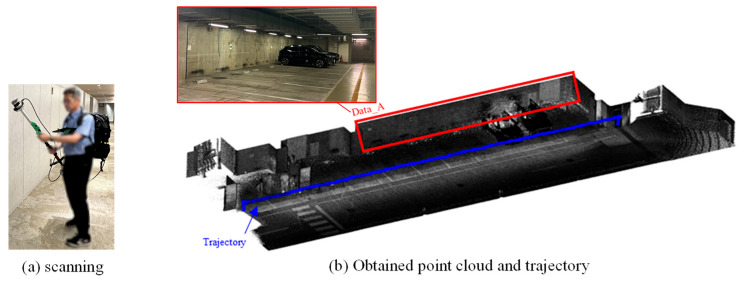
Scanning with Ouster LiDAR OS0.

The OS0 provides a horizontal field of view of 360° and a vertical field of view of 90°, making it susceptible to blind spots in the vertical direction. Therefore, as shown in Figure 7a, the scanner was mounted on a monopod and tilted at an angle so that the laser beams would strike the ceiling surface perpendicularly. The underground parking lot was scanned while walking at a normal pace. As shown in Figure 7b, the wall surface point cloud used in the experiment (referred to as Data_A) was acquired by walking in a straight line parallel to the target wall, maintaining a distance of approximately 6 m. The scanning duration was approximately 3 min. The raw dataset contained about 1 million points, which was reduced to approximately 0.5 million points through the sampling process. The smallest piece of building equipment identified in this dataset was a rectangular fire extinguisher sign measuring approximately 25 cm × 20 cm.

**Figure 8:**
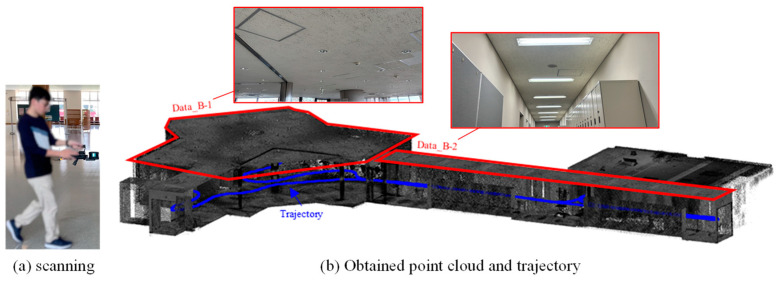
Scanning with Velodyne LiDAR VLP-16.

On the other hand, the VLP-16 provides a horizontal field of view of 360° and a vertical field of view of 30° and is likewise prone to blind spots in the vertical direction. To address this limitation, a Hovermap SLAM (Simultaneous Localization and Mapping) Unit by Emesent, equipped with the VLP-16, was held parallel to the floor, and the interior of a university building was scanned while walking at a normal pace, as shown in Figure 8a. By rotating the VLP-16 mounted at the tip of the Hovermap, uniform laser irradiation over a 360° × 360° area can be achieved. Figure 8b presents the point clouds used in the experiment: the ceiling surface of the elevator hall (Data_B-1) and that of the corridor (Data_B-2). The total scanning time was approximately 5 min. Data_B-1 originally contained approximately 4 million points, which were down sampled to 1.3 million, while Data_B-2 contained approximately 2 million points, reduced to 0.6 million after sampling. The smallest piece of building equipment identified in this dataset was a circular sprinkler with a diameter of approximately 10 cm.

### 4.2. Results of Intensity Correction

**Figure 9:**
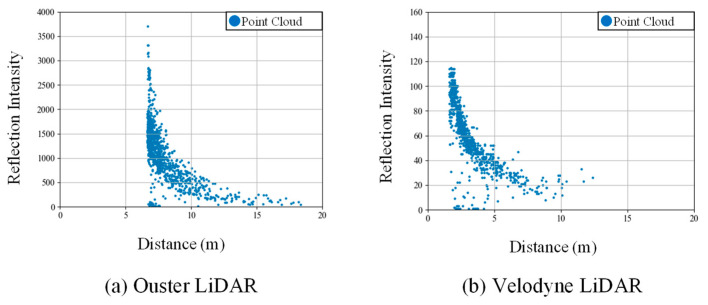
Reflection intensity distribution of Ouster and Velodyne LiDAR.

Figure 9 presents a comparison of the reflection intensity distributions from point clouds acquired using the OS0 and VLP-16 scanners. A study by Viswanath et al. [36] reported significant differences in the reflection intensity values obtained from these two types of scanners. Specifically, the reflection intensity values from Ouster LiDAR represent raw, uncalibrated data, whereas those from Velodyne LiDAR are pre-calibrated for distance and laser power, as documented in the manufacturer’s manual. The two scanners produce different intensity outputs due to the application of internal scaling. In this study, the scaled intensity values provided by each manufacturer were directly used in the experiments. As shown in Figure 9a, the data acquired with the OS0 exhibited a reflection intensity trend consistent with that reported in the literature. In contrast, the VLP-16 data, shown in Figure 9b, exhibited a decrease in reflection intensity with increasing distance. To address this discrepancy, reflection intensity correction was applied to both datasets using the method proposed by Wan et al. [34], as described in Section 3.3.

Table 3 summarizes the results of the reflection intensity correction. By comparing the initial and corrected reflection intensity values, it was observed that the standard deviation decreased after correction for both the Ouster and Velodyne LiDAR datasets, confirming the effectiveness of the correction. These results indicate that simultaneous correction of distance and incident angle using Equation (5) is applicable when scanning is performed while maintaining a constant distance between the scanning device and the target surface.

### 4.3. Results of Equipment Detection

**Figure 10:**
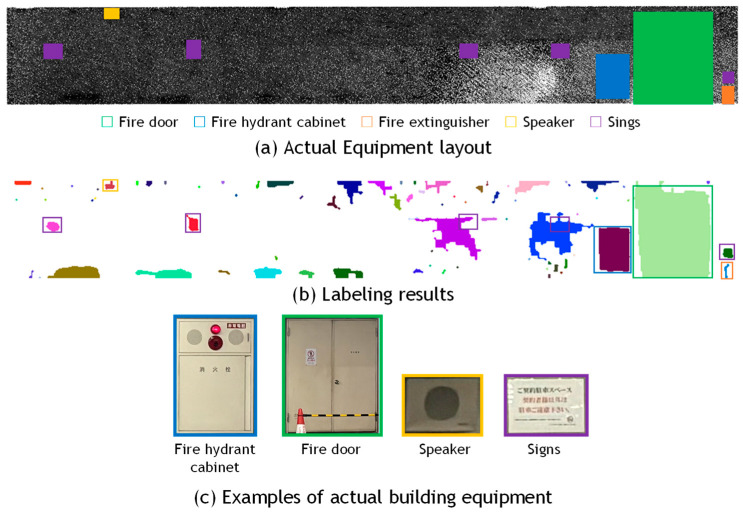
Visual evaluation of building equipment of Data_A.

**Figure 11:**
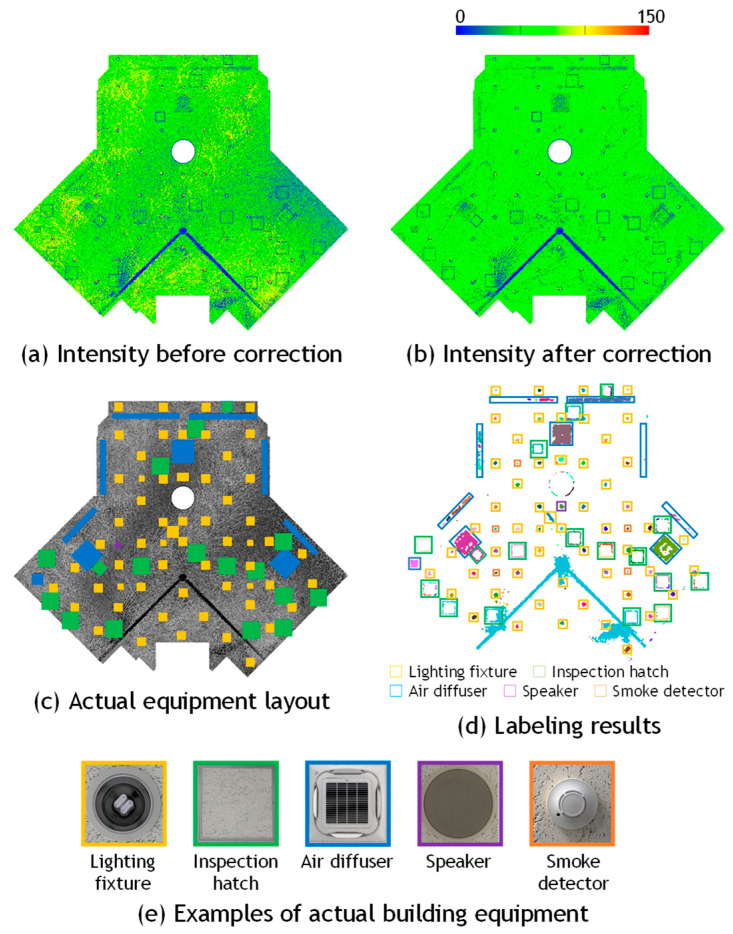
Visual evaluation of building equipment of Data_B1.

**Figure 12:**
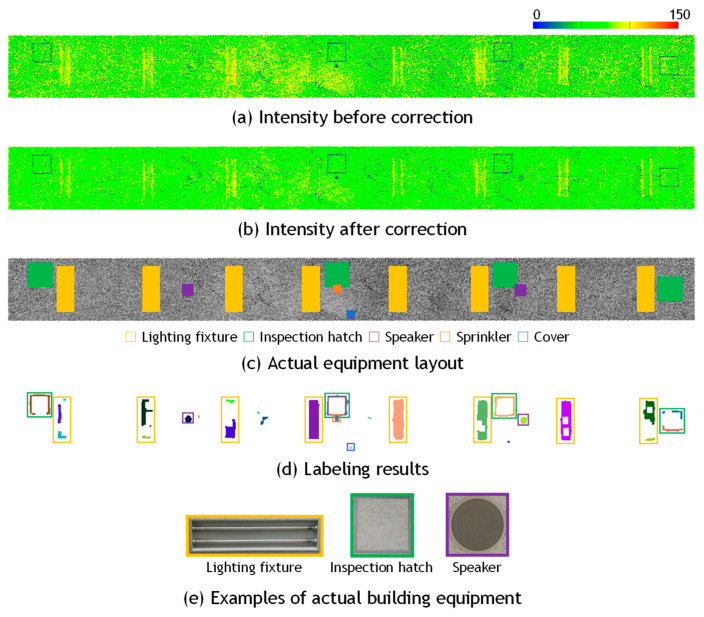
Visual evaluation of building equipment of Data_B2.

Figure 10, Figure 11 and Figure 12 present the results of building equipment detection. The detected equipment layout was visually compared with ground-truth images of the actual site. A piece of equipment was considered correctly detected if one or more labeled pixels were found at the corresponding location. Table 4 summarizes the detection results for each dataset. The threshold parameter was set to *α* = 1.0 for Data_A in Figure 10, and *α* = 2.0 for Data_B-1 in Figure 11, and Data_B-2 in Figure 12. The ground-truth data were manually created through direct visual inspection of the actual site and careful examination of multiple photographs taken at the location. 

This study summarizes five indicators—number of actual equipment, number of extracted regions, number of correct detections, number of missed detections, and number of over-detections—as shown in Table 4, allowing a more intuitive understanding of over-segmentation and merging tendencies in the extraction results. Each indicator is defined as described below, and the corresponding ratios in the table are calculated accordingly. Here, the number of actual equipment is denoted as $N_{eqp}$.

- Number of extracted regions: The total number of clusters consisting of one or more pixels detected in the image, denoted as $N_{ext}$. The ratio is calculated as $N_{ext}/N_{eqp}$ (%).
- Correct detection: The number of clusters consisting of one or more pixels that correctly correspond to actual equipment locations, denoted as $N_{crr}$. When one piece of equipment is divided into multiple regions, all such clusters are counted individually. The ratio is calculated as $N_{crr}/N_{eqp}$ (%). Note that this $N_{crr}$ is different from the standard metric True Positive (TP).
- Miss detection: The number of actual equipment objects for which no cluster was detected in the image, denoted as $N_{miss}$. The ratio is calculated as $N_{miss}/N_{eqp}$ (%). This $N_{miss}$ corresponds to the False Negative (FN).
- Over detection: The number of clusters consisting of one or more pixels that were incorrectly detected in regions where no actual equipment exists, denoted as $N_{over}$. The ratio is calculated as $N_{over}/N_{ext}$ (%). This $N_{over}$ corresponds to the False Positive (FP).

Overall, the number of extracted regions exceeded the actual number of equipment instances. The correct detection rates were relatively high for Data_B-1 and Data_B-2, which may be attributed to individual pieces of equipment being divided into multiple segments and detected as separate regions. Conversely, there were no missed detections. The degree of over-detection varied significantly depending on the dataset.

For Data_A, all nine pieces of equipment were successfully detected using the proposed method. However, in two cases, the equipment could not be detected independently and was extracted along with a broad surrounding area of the wall surface. This was likely due to the presence of parked vehicles in front of the signs during scanning, which obstructed the view and resulted in abnormally low reflection intensity values in the corresponding wall regions. The over-detection rate was 90.9%. One possible reason is that the wall surface in this dataset is made of concrete, which is easily subject to staining. Variations in reflection intensity caused by differing degrees of surface contamination may have led to portions of the stained areas being mistakenly detected as equipment candidates. In this example of the parking lot wall, as shown in the photograph in Figure 7b, several areas near the lower part of the wall, where exhaust gases from vehicles directly impinge, have become darkened due to surface discoloration. As illustrated in Figure 4a,b, the intensity values in these areas are extremely low, which has caused false detections in the results. In other parts of the wall, numerous minor stains caused by aging are also observed; however, these do not necessarily lead to false detections, and many regions are correctly recognized as wall surfaces without being affected by the stains. The proposed method assumes that the target surface is composed of a uniform material; however, this assumption was not satisfied in the case of stained concrete. As a result, it becomes challenging to accurately isolate only the equipment based solely on reflection intensity. For reference, the sign indicated by the purple frame on the right side of Figure 10c states that the parking space is reserved for contracted users.

**Figure 13:**
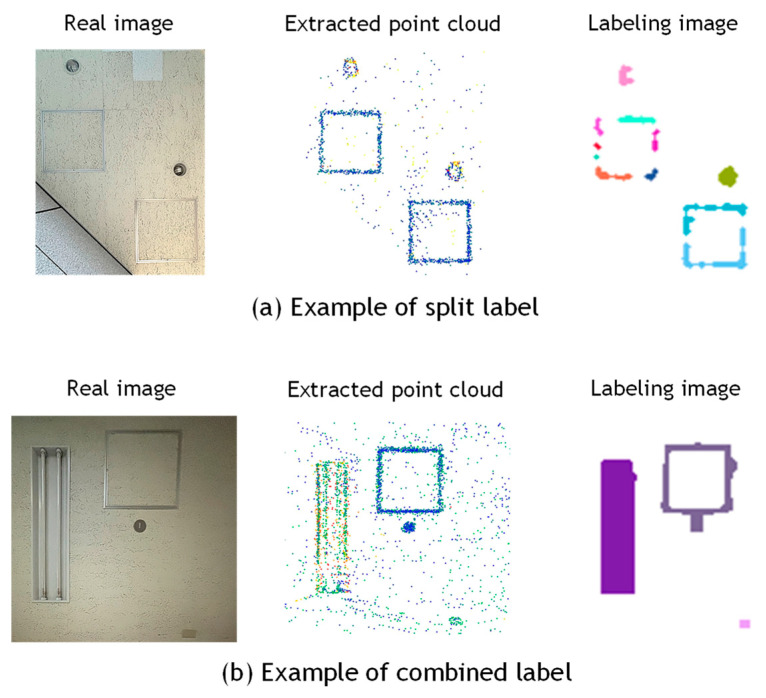
Examples of Region Splitting and Merging.

For Data_B-1 and Data_B-2, the over-detection rates were relatively low, at 27.9% and 17.9%, respectively. Since these indoor datasets closely adhered to the assumption of surface uniformity, favorable results were obtained. However, the number of detected regions was significantly larger than the actual number of equipment items, resulting in high correct detection rates of 197.7% and 175.0%, respectively. This discrepancy is attributed more to the image processing stage of the proposed method than to the reflection intensity correction. Even after applying morphological processing, the extracted pixels were not always sufficiently connected, causing a single piece of equipment to be segmented into multiple labeled regions. This issue was particularly evident in the case of ceiling inspection hatches, which consist of long and narrow metal frames, as shown in Figure 13a. Conversely, in cases where multiple pieces of equipment were located in close proximity, they were sometimes grouped and labeled as a single unit, as illustrated in Figure 13b. Another example is shown in Figure 11, where the method failed to correctly separate the metal support pillars of fire shutters and instead detected a broad region including adjacent equipment as a single entity. These results suggest the need to refine the criteria for connecting pixels identified as equipment candidates, to improve detection accuracy.

In this study, cases were observed where a single piece of equipment was divided into multiple detected regions (one-to-many), as well as cases where multiple pieces of equipment were merged into a single detected region (many-to-one). In other words, both over-segmentation and under-segmentation coexisted in the detection results. In such situations, conventional object-based evaluation metrics such as Precision and Recall cannot be directly applied. The standard evaluation indicators False Positive (FP) and False Negative (FN) correspond to over-detection and miss-detection in this study, respectively. However, True Positive (TP) does not necessarily coincide with correct detection. This is because, as mentioned above, a single piece of equipment may be divided into multiple clusters (one-to-many), or multiple pieces of equipment may be merged into one cluster (many-to-one). Considering these conditions, TP was conveniently defined in this study as follows: when a single piece of equipment was divided into multiple detected clusters, those clusters were integrated and counted as one; conversely, when multiple pieces of equipment were merged and detected as one region, they were counted individually. Based on this definition, Precision and Recall were calculated for wall and ceiling surfaces (Data_A, Data_B1, and Data_B2) together with FP and FN. As a result, FN was 0 for all datasets, and Recall was 1.0 in all cases, indicating that no equipment was missed. On the other hand, due to a relatively large number of FP, Precision showed lower values of 0.11 for Data_A, 0.64 for Data_B1, and 0.76 for Data_B2. These results confirm that, while Recall was perfect (1.0), Precision remained comparatively low, indicating a tendency toward over-detection in the results.

### 4.4. Verification of General Applicability Using Another MLS and TLS

**Figure 14:**
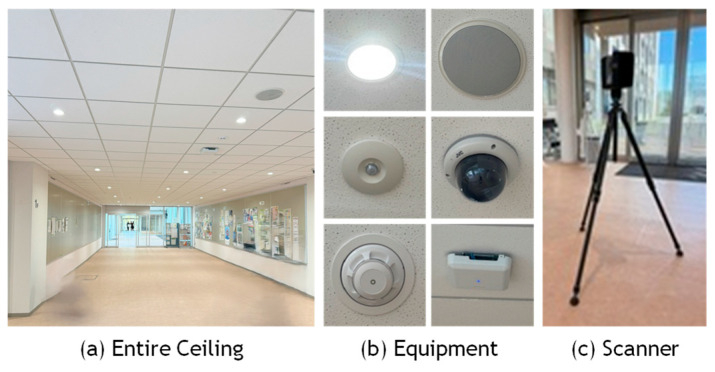
Test site for validation using TLS.

**Figure 15:**
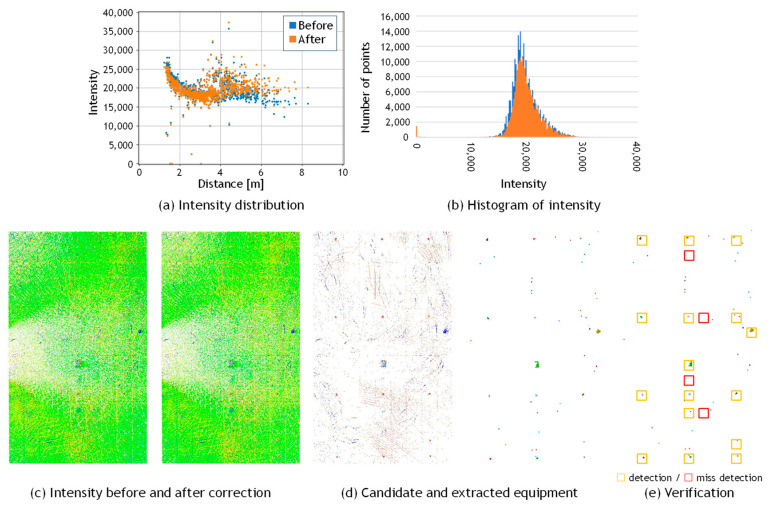
Results for MLS data of ceiling (Data_C).

To verify the general applicability of the proposed method, an additional experiment was conducted using a FARO Orbis laser scanner, which differs from the devices used in the previous experiments. Similarly to the Hovermap system, this scanner is a handheld unit equipped with a rotating mechanism that enables 360° × 360° omnidirectional scanning while walking. The maximum scanning range is 120 m, the scanning rate is 640,000 points per second, and the scanning accuracy is 5 mm. The experimental site was the ceiling of a university campus building, as shown in Figure 14a, which is different from the one used in Figure 8. As illustrated in Figure 14b, the ceiling contains a total of 20 pieces of equipment, including lighting fixtures, sprinklers, air diffusers, speakers, security cameras, and Wi-Fi routers. This dataset is referred to as Data_C (MLS). Figure 15a shows a scatter plot of reflection intensity before and after correction, and Figure 15b presents the corresponding histogram. Since the scanned area was relatively small and the maximum scanning distance was approximately 6 m, the decrease in reflection intensity with increasing distance was not clearly observed, and the correction effect could not be distinctly confirmed. However, the histogram in Figure 15b indicates that the corrected reflection intensities approximately followed a normal distribution. From the color representation of the reflection intensity shown in Figure 15c, no clear difference can be observed before and after the correction; however, the intensity values appear to be distributed approximately uniformly. The proposed detection method was then applied to this point cloud, with the threshold parameter set to *α* = 2.0. As a result, 16 pieces of equipment were successfully extracted, each forming a single cluster as shown in Figure 15d,e. In some cases, two lighting fixtures were split into two clusters. Three air diffusers and one sprinkler were not detected, most likely because both their color and material were similar to the ceiling background, resulting in no significant difference in reflection intensity. Although the effect of reflection-intensity correction could not be clearly confirmed for this dataset, satisfactory extraction results were obtained, demonstrating the general applicability of the proposed method.

**Figure 16:**
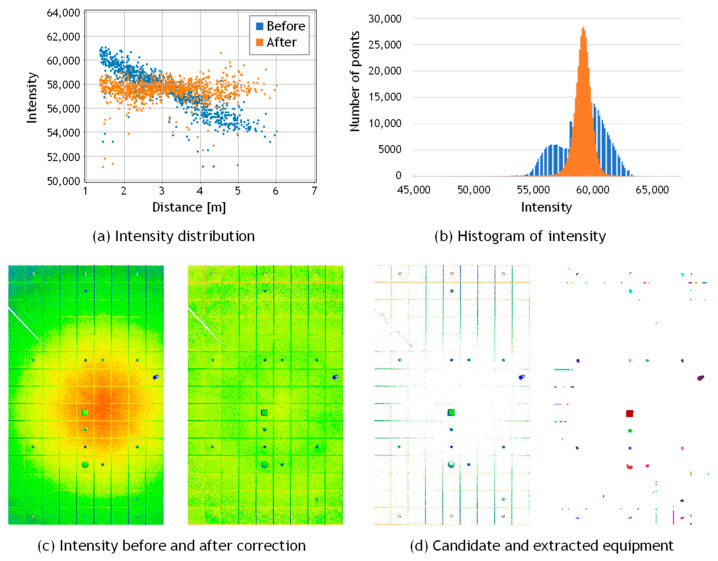
Results for TLS data of ceiling (Data_C).

Here we present the experimental results using point cloud data acquired by a terrestrial laser scanner (TLS) to compare and validate the results obtained from mobile laser scanning (MLS). The test site was a ceiling surface on a university campus which is the same as in Figure 14a. The TLS experiment is referred to as Data_C (TLS), and the results are shown in Figure 16 and Table 4. The point cloud was acquired using a FARO Focus3D scanner, as shown in Figure 14c, with data captured from a single scanning position. The point cloud was down sampled at approximately 1 cm intervals. Figure 16a shows a scatter plot of reflection intensity before and after correction, and Figure 16b presents the corresponding histogram. Since the scanner was positioned vertically and scanned a flat ceiling surface, the incident angle was constant when the distance was constant. Figure 16c visualizes the reflection intensity using color coding. It is clearly observed that the reflection intensity is highest directly beneath the scanner and gradually decreases in a concentric pattern outward from the center. Although this is an empirical observation, these results suggest that the correction effect for TLS is greater than that for MLS, with the corrected reflection intensity approaching a nearly constant value. Furthermore, as shown in Figure 16d, all equipment items were detected as distinct clusters, without being divided into multiple regions or merged with adjacent equipment. The threshold parameter set to *α* = 2.0. However, the ceiling surface was composed of multiple panels, and the point clouds corresponding to the gaps between these panels were mistakenly detected as equipment, resulting in a relatively high over-detection rate. This example also corresponds to a case where background uniformity is not satisfied, similar to the wall example discussed in Section 4.3, and false detections have likewise occurred in this region. Despite the difference in scanners, the overall detection behavior was consistent with the MLS results. These findings confirm that the proposed method is applicable not only to MLS data but also to TLS data.

### 4.5. Discussion

**Figure 17:**
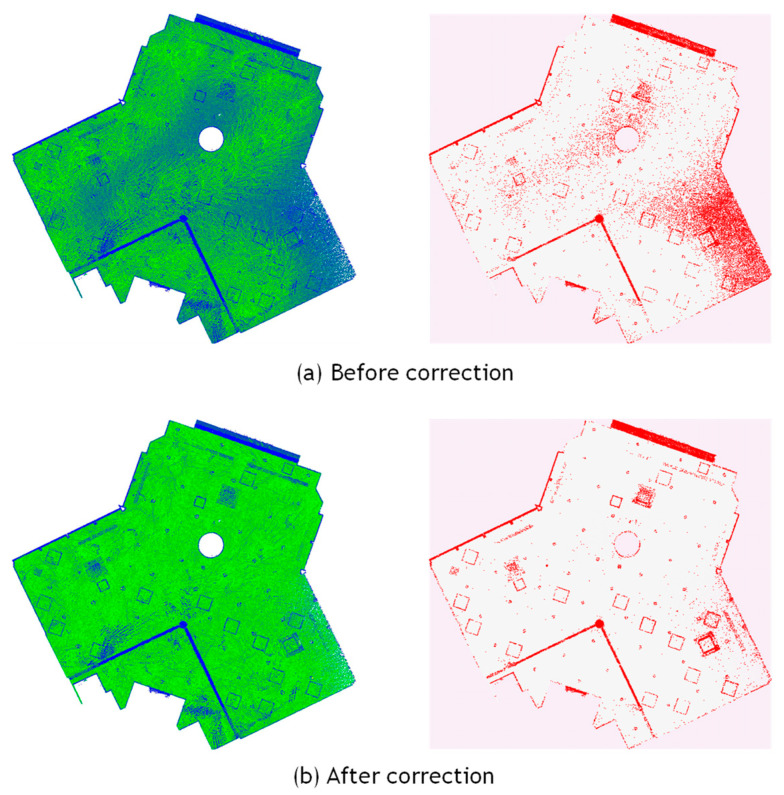
Comparison of laser intensity and equipment candidate extraction before and after correction.

Regarding the comparison with other methods, it is evident from the graph that applying a simple thresholding approach to uncorrected intensity values makes object detection difficult. In MLS scanning, even at the same location on a background surface such as a wall or ceiling, the intensity values exhibit large variations between points scanned vertically from a short distance and those scanned obliquely from a farther position. Consequently, setting a threshold on such uncorrected intensity data cannot properly extract equipment regions. In contrast, by first applying an appropriate intensity correction to normalize the background wall and ceiling values and then setting a threshold to identify the background surface, points deviating from the normal distribution of intensity values can be effectively extracted as equipment candidates. Figure 17 shows the reflection intensity values before and after correction, along with the extraction results of equipment candidate points. The measurement target is the same ceiling surface as in Data_B1. As shown in Figure 17a, the reflection intensity values on the ceiling surface before correction exhibit large variations caused by measurement distance and incidence angle. As a result, some equipment was not extracted, and non-equipment regions were frequently detected by mistake. In contrast, as shown in Figure 17b, after correction, the reflection intensity values on the ceiling surface become closer to a uniform level, making it easier to visually recognize equipment from the intensity image. Furthermore, subsequent thresholding appropriately extracted equipment candidates and reduced false detections. In this example as well, the threshold was set to *α* = 2.0.

One of the advantages of the proposed method lies in the use of Mobile Laser Scanning (MLS). Compared with the conventional Terrestrial Laser Scanning (TLS), MLS enables more efficient scanning over larger areas. In the geometric detection method for TLS point cloud proposed by Akiyama et al. [14], the scanner was placed at approximately 10 m intervals for data acquisition, which resulted in a substantial increase in scanning time. Moreover, the study reported that the detection accuracy of equipment decreases when the scanning distance exceeds 5 m. In contrast, the proposed method achieves a significant reduction in scanning time by employing MLS. By designing densely spaced scan trajectories and conducting scanning from short distances to the background surfaces and equipment, a sufficient point density can be ensured. As a result, the proposed approach is less affected by the decline in detection rate associated with longer scanning distances. Although the geometric accuracy of MLS is on the order of a few centimeters—an order of magnitude lower than that of TLS—the use of intensity information enables reliable detection of thin, planar structures such as inspection hatches, which are difficult to identify using purely geometric methods.

Furthermore, another advantage is that it produces fewer miss detections compared to conventional object detection methods based on point clouds or images. In the point cloud data used in this experiment, more than 100 pieces of equipment were detected with almost no omission, and this result is considered useful for subsequent processes such as classification and 3D modeling. In this experiment, the point cloud was uniformly down sampled at a 2 cm pitch in the binary imaging process; nevertheless, even from this data, a small sprinkler with a diameter of approximately 10 cm, installed on a ceiling about 3 m above the ground, was successfully extracted. This device was represented by approximately 20 pixels in the binary image. As for even smaller equipment, sprinkler heads can be mentioned, some of which have a diameter as small as 3 cm. However, at the current imaging resolution, such equipment is represented by very few pixels, making detection difficult. To target such small-sized devices, it will be necessary to increase the imaging resolution to approximately 1 cm or higher.

Regarding computation time, even for Data_B, which has the largest surface area and the highest number of points among the datasets used in this study, the total processing time was only a few seconds, demonstrating high computational efficiency. All procedures were implemented as batch processing and executed collectively after point cloud generation following the scanning. The implementation and experiments were conducted on a standard PC environment running Windows 10, an Intel Core i7 CPU, and 64 GB of RAM. The code was written in C++.

A limitation of the proposed method arises in cases where there is variation in the reflection intensity of the background, as illustrated in the example shown in Figure 10 and Figure 16. When differences in background color or material exist, such as stains on the wall or deteriorated paint, variations in reflection intensity may cause non-equipment regions to be mistakenly extracted. A potential solution to this issue is to perform a more comprehensive analysis by combining both image data and reflection intensity, rather than relying on either one in isolation.

Future challenges lie in the classification of the extracted equipment. In recent years, in the field of object recognition, methods that simultaneously perform object detection and classification, such as YOLO [37], have become mainstream. However, YOLO was originally developed with the aim of achieving both high processing speed and recognition accuracy. On the other hand, in applications such as this study, where processing speed is not a primary requirement, it is an important direction for future research to consider alternative approaches that prioritize accuracy even at the expense of longer processing time. For example, after performing omission-free object detection using the proposed method based on reflectance intensity, it would be effective to generate orthophotos from the images acquired simultaneously with the 3D point cloud. In such orthophotos, objects are aligned with vertical and horizontal axes. Consequently, combining point cloud and image data is expected to enhance the object detection results compared to using point clouds alone, and to enable more accurate classification. Furthermore, when the image resolution and quality is insufficient, the introduction of super-resolution techniques [38] or appropriate preprocessing, such as motion blur removal [39], can be expected to further enhance the accuracy of object recognition.

In addition, for the polynomial fitting between distance and intensity, we aim to reduce the influence of occlusions, surface stains, and vehicles by applying robust regression methods such as RANSAC or Huber loss. The improvement in extraction performance achieved by these robust approaches will be evaluated in comparison with the currently adopted ordinary least squares method.

## 5. Conclusions

In this study, we proposed a method for detecting building equipment based on reflection intensity correction, with the objective of constructing detailed BIMs from point clouds acquired via MLS. In the proposed approach, we first demonstrated that the effects of the scanning distance and incident angle embedded in the laser reflection intensity can be effectively eliminated from ceiling and wall point clouds obtained by MLS, using polynomial approximation. We verified that the reflection intensity of flat surfaces made of uniform materials, such as ceilings and walls, can be corrected to approximate a constant value. Furthermore, we demonstrated that the corrected reflection intensity enables the detection of building equipment with almost no omission, through a combination of thresholding and morphological processing. Through various experiments and validations, the results of this study have been demonstrated to contribute effectively to the construction of detailed Building Information Models (BIMs) from mobile laser-scanned data.

Future work will focus on addressing over-detection of equipment regions, as well as handling cases where a single piece of equipment is fragmented into multiple components. We plan to introduce post-processing procedures for region merging and splitting. Specifically, incorporating 3D shape connectivity, planar distance-based region integration on the projected surface, or least-squares fitting of geometric primitives such as rectangles and circles is expected to mitigate over-segmentation and under-segmentation. Furthermore, detection performance will be assessed for each equipment category (e.g., lights, sprinklers, speakers, and signs) to identify which classes benefit most from the use of intensity information. Moreover, the use of recently developed hyperspectral LiDAR systems may enable more accurate detection and classification of building equipment by considering the wavelength-dependent characteristics of reflectance [32]. In addition, we plan to compare the proposed correction method with other existing approaches and explore its applicability to buildings with non-flat or more complex geometries. We also focus on integrating corrected intensity data with orthophoto color information to further reduce false detections caused by stains and illumination variations. Although this study focused on equipment detection from pre-acquired LiDAR data, future work may also consider data compression and communication issues that are essential for practical and large-scale applications [40,41].

## Figures and Tables

**Table 1:** Specification of scanners.

Instrument	OS0 (Ouster)	VLP-16 (Velodyne)
Weight	430 g	830 g
Maximum distance	75 m (80% reflectivity) 35 m (10% reflectivity)	100 m
Accuracy	±2.5 cm (Lambert Target) ±5.0 cm (Retroreflective Target)	±3 cm
Field of view	360° × 90°	360° × 30°
Data acquisition rate	5.2 M points/second	0.3 M points/second

**Table 2:** Overview of our test site.

	Data_A	Data_B1	Data_B2	Data_C (MLS)	Data_C (TLS)
Scanner	Ouster OS0	Emesent HoverMap	Emesent HoverMap	FARO Orbis	FARO Focus3D
Scanning site	Wall of underground parking lot	Ceiling of building	Ceiling of building	Ceiling of building	Ceiling of building
Scanning area (m^2^)	42.3	125.2	44.6	98.0	98.0
Average scanning distance (m)	8.0	3.1	2.5	3.4	3.1
Number of points before/after down sampling (upper/lower row)	42,561,880 495,598	27,356,221 1,286,807	27,356,221 588,432	100,246,931 517,217	43,865,406 393,200
Number of equipment by class	Fire door (1) Fire hydrant cabinet (1) Fire extinguisher (1) Speaker (1) Sign (5)	Lighting fixture (56) Inspection hatch (16) Air diffuser (Square) (3) Air diffuser (Slot) (6) Speaker (1) Smoke detector (5)	Lighting fixture (8) Inspection hatch (4) Speaker (2) Sprinkler (1) Cover (1)	Lighting fixture (12) Sprinkler (2) Air diffuser (3) Speaker (1) Security camera (1) Wi-Fi router (1)	Lighting fixture (12) Sprinkler (2) Air diffuser (3) Speaker (1) Security camera (1) Wi-Fi router (1)

**Table 3:** Results of reflection intensity correction.

Scanner	Target Area		Average	Standard Deviation
Ouster LiDAR	Wall of underground parking (Data_A)	Before	1101.0	605.5
		After	904.9	309.3
Velodyne LiDAR	Ceiling of elevator hall (Data_B-1)	Before	66.4	27.2
		After	61.9	16.6
	Ceiling of the hallway (Data_B-2)	Before	78.7	22.5
		After	76.0	16.6

**Table 4:** Results of equipment detection based on our metric.

	Data_A		Data_B-1		Data_B-2		Data_C (MLS)		Data_C (TLS)	
Number of equipment	9	-	87	-	16	-	20	-	20	-
Number of extracted regions	77	855.6%	172	197.7%	28	175.0%	48	240.0%	67	335.0%
Correct detection	9	100.0%	124	142.5%	23	143.8%	18	90.0%	20	100.0%
Miss detection	0	0.0%	0	0.0%	0	0.0%	4	20.0%	0	0.0%
Over detection	70	90.9%	48	27.9%	5	17.9%	30	62.5%	47	70.2%

## Data Availability

The datasets generated and/or analyzed during the current study are available from the corresponding author on reasonable request. The implementation code will be shared after project completion, subject to institutional and collaborative agreements.
